# A Pyrosequencing Investigation of Differences in the Feline Subgingival Microbiota in Health, Gingivitis and Mild Periodontitis

**DOI:** 10.1371/journal.pone.0136986

**Published:** 2015-11-25

**Authors:** Stephen Harris, Julie Croft, Ciaran O’Flynn, Oliver Deusch, Alison Colyer, Judi Allsopp, Lisa Milella, Ian J. Davis

**Affiliations:** 1 The WALTHAM Centre for Pet Nutrition, Mars Petcare UK, Leicestershire, United Kingdom; 2 The Veterinary Dental Surgery, Surrey, United Kingdom; University Hospital of the Albert-Ludwigs-University Freiburg, GERMANY

## Abstract

Periodontitis is the most frequently diagnosed health problem in cats yet little is known about the bacterial species important for the disease. The objective of this study was to identify bacterial species associated with health, gingivitis or mild periodontitis (<25% attachment loss) in feline plaque. Knowledge of these species is a first step in understanding the potential for improving oral health of cats via dietary interventions that alter the proportions of influential species. Subgingival plaque samples were collected from 92 cats with healthy gingiva, gingivitis or mild periodontitis. Pyrosequencing of the V1-V3 region of the 16S rDNA from these plaque samples generated more than one million reads and identified a total of 267 operational taxonomic units after bioinformatic and statistical analysis. *Porphyromonas* was the most abundant genus in all gingival health categories, particularly in health along with *Moraxella* and Fusobacteria. The Peptostreptococcaceae were the most abundant family in gingivitis and mild periodontitis. Logistic regression analysis identified species from various genera that were significantly associated with health, gingivitis or mild periodontitis. The species identified were very similar to those observed in canine plaque in the corresponding health and disease states. Such similarities were not observed between cat and human at the bacterial species level but with disease progression similarities did emerge at the phylum level. This suggests that interventions targeted at human pathogenic species will not be effective for use in cats but there is more potential for commonalities in interventions for cats and dogs.

## Introduction

Periodontitis is the most commonly diagnosed health problem in cats [1]. The reported incidence levels vary from study to study but are generally high. For example, a radiographic study by Lommer and Verstraete [2] recorded that 72% of 147 cats examined had some degree of periodontitis. Similarly a study of a colony of 109 cats by Girard *et al*., [3] found that 98.2% had some degree of periodontal bone loss. Despite the prevalence of periodontal disease in domestic cats, our current understanding of the possible aetiological agents of the disease is limited, and there is little information documenting the bacteria found in the oral cavity. Many of the early feline oral bacteriology studies concentrated on isolation of bacteria from cat bite wounds [4]. The first study to survey the microbial flora directly associated with feline periodontal disease was carried out by Mallonee *et al*., [5]. This culture based investigation analysed subgingival plaque samples from 32 cats at various stages of periodontal disease. Cats were sampled at a least-affected site and a most-affected site for comparison. A greater number of anaerobic Gram-negative rods were identified at most-affected sites in those individuals with higher gingival index scores. With increasing severity of periodontal disease, increasing numbers of black pigmented *Bacteroides* and *Peptostreptococcus* anaerobes were isolated. A more recent study by Sturgeon *et al*., [6] made use of culture- independent next generation sequencing to identify the bacteria species common in healthy cat plaque. In this study of 11 cats, the most prevalent genus-level phylotypes were: unclassified Pasteurellaceae (18.7%), Moraxella (10.9%), Thermomonas (6.9%), an unclassified Comamonadaceae (5.6%), Neisseria (4.9%), an unclassified Moraxellaceae (4.4%), and Pasteurella (4.3%). Other studies into cat oral bacteria have focussed on looking for the presence of a small number of defined species [7, 8]. As such, the objective of this 16S pyrosequencing investigation was to elucidate differences in the oral microbiota in feline plaque in health, gingivitis and mild periodontitis in a culture-independent manner. A recent study by Dewhirst *et al*., [9] has facilitated the process through the development a database of 16S rDNA based taxonomy that represents 171 feline oral taxa. The resulting feline oral microbiome curated taxonomy and 16S rRNA gene reference set allowed the precise definition of bacterial taxa and was used along with the canine oral microbiome reference set [10] to annotate species within this study. The knowledge gained of the species prevalent in the health and disease states is a step towards understanding the potential for dietary interventions to alter the proportions of these species and improve oral health of cats.

## Materials and Methods

### Ethics Statement, Sampling Strategy and Study Cohort

The study was approved by the WALTHAM Centre for Pet Nutrition ethical review committee, owner consent was obtained and an owner survey was completed for all cats included in the study. The study cohort comprised client owned pet cats presented at four veterinary referral dental clinics in the UK. This cross-sectional study included only cats under anaesthetic for routine dental treatment or treatment for non-periodontal complications, e.g. fractured teeth, or other non-infectious conditions were screened for inclusion in the study to allow the collection of healthy samples. No cats were anaesthetised solely for the collection of plaque samples. To ensure consistency of dental assessments, scoring and subgingival plaque sampling, all veterinarians were trained by a veterinary dentist. The periodontal health status of each cat was determined following a modified Wiggs & Lobprise scoring system [11]. Plaque samples were taken from cats regarded as having healthy teeth and gums, gingivitis or mild periodontitis (<25% attachment loss). Since not all teeth in a mouth were in the same health state a system was put in place to determine the predominating health state for each mouth, and samples were only collected from that health state. In brief, a mouth was considered healthy if most or all the teeth had a gingivitis score of 0 and no teeth had a score of more than 2. Only teeth with a gingivitis score of 0 were sampled for the healthy group. If a mouth had teeth with gingivitis scores of mostly 1 to 3 but no periodontitis it was considered part of the gingivitis group (no gingivitis 4 teeth were observed). Only teeth with gingivitis scores 1 to 3 were sampled in this group. Finally, if a mouth had any teeth with mild periodontitis it was considered part of the mild periodontitis group. Only teeth with mild periodontitis were sampled from in this case. The gingivitis score of each sampled tooth was noted along with periodontal pocket depths for the mild periodontitis samples. Sub gingival plaque was collected using a sterile periodontal probe and then placed in 300μl TE buffer (50 mM Tris pH 7.6, 1 mM EDTA pH 8.0 & 0.5% Tween 20) prior to storage at -20°C. Information on cat age, and sex was also collected.

Cats were excluded from the study if they had: 1) Significant veterinary oral care within the preceding 3 months; 2) Regular dental care at home i.e. cats whose teeth are regularly brushed; 3) Systemic or oral antibiotic treatment at any time during the previous 3 months and 4) Evidence of any extra-oral bacterial infections in the past month. Pure breed cats were also excluded; there is anecdotal evidence that certain pure breeds are predisposed to periodontitis, which suggests that there may be breed specific disease aetiology that could confound the results.

### DNA extraction and amplification of 16S rDNA

Bacterial DNA was extracted once all plaque samples had been collected. DNA was extracted from the plaque samples using an Epicentre Masterpure Gram Positive DNA Purification Kit, according to the manufacturer’s instructions with an additional overnight lysis. Plaque samples were centrifuged at 5000 x g for 10 minutes and the cell pellet resuspended in 150μl of TE buffer. Following vortexing, 1 μl Ready-Lyse Lysozyme (Epicentre, UK) was added and the lysis mix incubated overnight at 37°C for 18hrs overnight. After DNA extraction the DNA pellet was suspended in TE buffer (10mM Tris-Cl and 0.5 mM pH 9.0 EDTA) and quantified and the purity ascertained using a NanoDrop ND1000 spectrophotometer (NanoDrop Technologies Inc). The V1-V3 region of the 16S rDNA was amplified from subgingival plaque DNA extractions using Extensor Hi-Fidelity PCR Enzyme Mix (AB-0792, Thermo, UK) in a 96-well format. A mix of two universal forward primers was used; FLX 27FYM (*CGTATCGCCTCCCTCGCGCCATCAG* **AGAGTTTGATYMTGGCTCAG**) at 9.5pmol/μl and FLX 27F Bif (*CGTATCGCCTCCCTCGCGCCATCAG* **AGGGTTCGATTCTGGCTCAG**) at 0.5pmol/μl (where italics represent FLX Titanium Primer A and bold represents 16S sDNA primer sequence). The latter was included to ensure representation of the genus *Bifidobacter*; a lower concentration was chosen due to the low representation of this genus in previous studies of canine plaque. The DNA was to be sequenced from the reverse primer, thus 20 different 7mer MID tags were included in the reverse primer (*CTATGCGCCTTGCCAGCCCGCTCAGX*XXXXXX**TYACCGCGG CTGCTGG**) where italics represent FLX Titanium Primer B, X represents MID sequence and bold represents 16S sDNA reverse primer sequence.

### Library preparation

Library preparation and sequencing was all performed by Eurofins. The 16S rDNA amplicons were purified and quantified then pooled into groups of 60 samples prior to Emulsion PCR. Libraries were then sequenced on a Roche Sequencer FLX Titanium System™ using the FLX Titanium B primer only with a target of ~ 15,000 unidirectional reads per sample.

### Sequence processing and analysis

The standard flowgram files (SFF) for each of the 92 samples were initially filtered by selecting reads with at least 360 flows and truncating long reads to 720 flows. Reads were filtered and de-noised using AmpliconNoise software (v1.21 [12, 13]). For the initial filtering step, reads were truncated when flow signals dropped below 0.7. A maximum of 20,000 reads per sample were used with exception of a few samples due to the computational demands of the de-noising algorithm. Subsequently reads were de-noised in three stages; 1) Pyronoise to remove noise from flowgrams resulting from 454 sequencing errors (Pyronoise M parameters -s 60, -c 0.01), 2) Seqnoise to remove errors resulting from PCR amplification (SeqNoiseM parameters -s 25, -c 0.08), 3) Perseus to detect and remove chimeras resulting from PCR recombination.

The de-noised sequences were then clustered using QIIME v1.7 [14].The QIIME script pick otus.py, which utilises Uclust v1.2.22q [15] was used to cluster sequences with >98% identity. Uclust was run with modified parameters, with gap opening penalty set to 2.0 and gap extension penalty set to 1.0 and–A flag to ensure optimum alignment [14]. Representative sequences of all Operational Taxonomic Units (OTUs) that passed the filtering criteria for sequence abundance (see statistical analysis section) across health states were searched against a 16S database using NCBI-BLAST v2.2.28+ [16] with the parameters “-penalty -5 -reward 4 -gapopen 5 -gapextend 5”. The database consisted of the Silva SSU database release 119 [17] which included 460 16S sequences from the Canine Oral Microbiome Database (COMD; [10] accession numbers JN713151-JN713566 & KF030193-KF030235). The database was supplemented with 248 recently published 16S sequences from the Feline Oral Microbiome Database (FOMD; [9]; accession numbers KM461942–KM462187).

Sequence annotation against a reference database can require a trade-off between multiple factors: the sequence identity of the query sequence to the hit in the database, the annotation detail of the hit and the consistency of the annotations for the hits above a certain identity threshold. We applied the following strategy for annotation and selection of full length representative sequences: if the OTU aligned to a subject from the FOMD database with ≥99% identity for ≥99% query coverage the assignment was accepted; if these criteria were not met the same criteria were applied against the COMD database; if these criteria were not met the subject with best hit (bit score) was used for taxonomic assignment. In some exceptions where the best hit subject was poorly annotated or inconsistent with near best hits, discretion was used to assign the OTU to the next best hit. Annotations below 98% and 95% identity were reduced to genus and family level annotation, respectively, to indicate a lack of confidence for more detailed taxonomy assignments.

A multiple sequence alignment (MSA) was constructed by aligning each reference sequence to the Greengenes [18] core set (revision May 2011) with PyNAST [14] using the script align seqs.py of the QIIME pipeline [19]. The MSA was filtered using the filter alignment.py script of the QIIME pipeline. A maximum likelihood tree of 1000 bootstrap replicates was inferred with FastTree v2.1.7 [20]. A GTR model of nucleotide substitution was chosen and the proportion of invariant sites was estimated from the data. Evolutionary rates were approximated by a discrete gamma model of 32 categories. The tree was visualised and combined with abundance and significance data in iTOL [21, 22].

A second tree with a reduced number of taxa was inferred at the genus level. For this purpose all species of the same genus were collated into a single taxon. The 16S sequence of the most abundant species of that genus was used for tree inference using the methods described above. If no genus information was present, taxa forming a clade in the full tree were grouped together and the new taxon was named e.g. “Actinomyces clade A”. Abundance information was added up for all members of each summarised taxon and plotted on the tree using iTOL. The tree was complemented with information on the number of original taxa summarised and the number of significant taxa. See S1 Table on which taxa were grouped together.

### Statistical analysis

Health and disease associations: OTUs were classified in a single group of “rare” taxa if either they were present in all health status groups at an average proportion below 0.05% or were present in less than two samples. The 0.05% cut-off was selected based on statistical analysis of data from mock communities containing 17 known species sequenced on five separate 454 runs. The mock communities were analysed for presence and absence of species using a false positive rate of 0.3% (i.e. finding species that were not included in the mock community) and false negative rate of 1.7% (i.e. the failure to identify the species that were known to be present) and aimed for a coefficient of variation of <20% (data not shown). The most abundant OTUs were then analysed using logistic regression analyses (Generalised linear model with a binomial distribution and logit link) for proportions, using the count of an OTU out of the total number of sequences, with health status included as a fixed effect. To allow the methods to enable estimation with many zero counts, 2 counts were added to each OTU count and 4 counts were added to the total count prior to analyses, analogous to adding 2 successes and 2 failures [23]. In addition, as the data were of very low proportions ~0.1%, a permutation test (1000 permutations) was used to allow for deviations from the logistic regression analysis assumptions. The permutation test *p*-values were then adjusted according to the false discovery method of [24] to allow for the increased likelihood of false positives when analysing the 267 OTUs which remained after rare classification.

Association with gingivitis score: owing to the system employed for sample collection, healthy samples had an average gingivitis score of 0 and gingivitis and mild periodontitis had an average gingivitis score of between 1 and 3. Individual OTUs were analysed univariately by logistic regression analyses (generalised linear model with a binomial distribution and logit link) for proportions, using the count for the OTU out of the total number of sequences, with the average gingivitis score for the sampled teeth as a continuous fixed effect. To enable robust estimation with many zero counts, 2 counts were added to each OTU count and 4 counts were added to the total prior to analyses. In addition, due to the low proportions, permutation tests were used to test the assumptions of the logistic regression analysis. To adjust for this multiplicity effect, the permutation *p*-values were then adjusted according to the false discovery method of [24].

Principal component analysis (PCA) was performed on the log_10_ proportions (after 2 counts were added to each OTU count and 4 counts were added to the total) to determine if variability of the most abundant OTUs was associated with health status, age and gender. The principle coordinates analyses (PCoA) ordination was performed using the *dudi*.*pco()* function from *R* package ade4 v1.6.2 (Dray and Dufour, 2007) using the Jensen-Shannon divergence (JSD) as a distance measure, as implemented in *R* by Arumugam *et al*., (2011). The PCoA vectors were plotted with shape areas weighted by OTU relative abundance using *R* package ggplot2 v1.0.0 (Wickman, 2009).

Gram-stain status: the OTUs, excluding the rare group, were classified as Gram positive or Gram negative based on literature searches using the genus name. The number of Gram positive sequences, out of the total number of sequences, were then analysed by logistic regression for proportions (allowing for estimation of over dispersion) with health status as a fixed effect.

Oxygen requirement: the non-rare OTUs were classified as aerobic, anaerobic or facultative anaerobic based on literature searches using the genus name. The number of aerobic, anaerobic and facultative anaerobic sequences, out of the total number of sequences, were then analysed (separately) by logistic regression for proportions (allowing for estimation of over dispersion) with health status as a fixed effect.

Shannon diversity index: a linear model was used to analyse the indexes, with health status as a fixed effect.

Species richness: a linear model was used to analyse the number of OTUs identified (including the rare sequences), with health status as a fixed effect, the total number of sequences as a covariate (to adjust for the differing number of sequences between samples).

Univariate statistical analyses were performed in GenStat v14.1 software and multivariate analyses in R v3.0.1.

## Results

### Study Cohort

Subgingival plaque bacterial communities were sampled from a total of 92 cats; 20 with healthy gingiva, 50 with gingivitis and 22 with mild periodontitis (PD1). The ages profile of the cats in the different health state groups was similar with the average ages of the healthy, gingivitis and early periodontitis groups being 4.3 (SD 3.6), 5.1 (SD.4.4) and 5.3 (SD 4.1) respectively (S2 Table). The healthy and mild periodontitis groups were well balanced for sex, with 9 males and 11 females in the healthy group and 13 males and 9 females in the mild periodontitis group. The gingivitis group was dominated by males containing 32 males and only 18 females. Other sample associated metadata is also captured in S2 Table.

### Sequence quality

The 92 subgingival plaque samples were analysed by 454-pyrosequenicng of the 3’ end of the V1-V3 region and a total of 1,774,113 sequence reads were obtained that passed the sequencing provider’s initial sequence quality filter. The sequence data is available on the European Nucleotide Archive under project accession number PRJEB9896, samples ERS792402- ERS792492. After Pyronoise, Seqnoise and chimera removal using Perseus the number of sequence reads was reduced to 1,112,543. The final number of sequence reads per sample ranged from 4,989 to 15,941 with a median number of reads of 12,830, 12,892 and 13,010 from healthy, gingivitis and mild periodontitis samples respectively. The sequence depths were analysed by ANOVA with health status as a fixed factor. There was no significant difference in the average sequence depth between health states, *p* = 0.829.

### Bacterial composition of feline plaque

The resulting 1,112,543 sequences were assigned to 9,638 operational taxonomic units (OTUs) using U-clust within QIIME and a cut-off of ≥98% sequence identity. OTUs were classed and grouped as rare if either they were present in each health status group at an average proportion below 0.05% or were present in less than two samples (see methods for rationale). This reduced the number of OTUs analysed to 267 plus the rare group. Taxonomic assignment of each of the 267 OTUs resulted in 227 (85%) mapping at ≥98% identity (species level) to the combined FOMD, COMD, Silva database. A further 25 (9%) of the OTUs mapped at between ≥96% and <98% (genus level). The remaining 15 (6%) mapped at <96% identity. The majority of the sequences belonged to seven phyla; Firmicutes (29.96%), Bacteroidetes (21.78%), Proteobacteria (16.67%), and Actinobacteria (8.22%), Spirochaetae (7.36%), Fusobacteria (3.62%) and Chlorobi (3.18%). There were also a further 4 phyla that were identified; Synergistetes (1.69%), Chloroflexi (1.31%), SR1 (0.95%), and TM7 (0.56%). The rare group accounted for the remaining 4.7% of the sequence reads.

**Fig 1:**
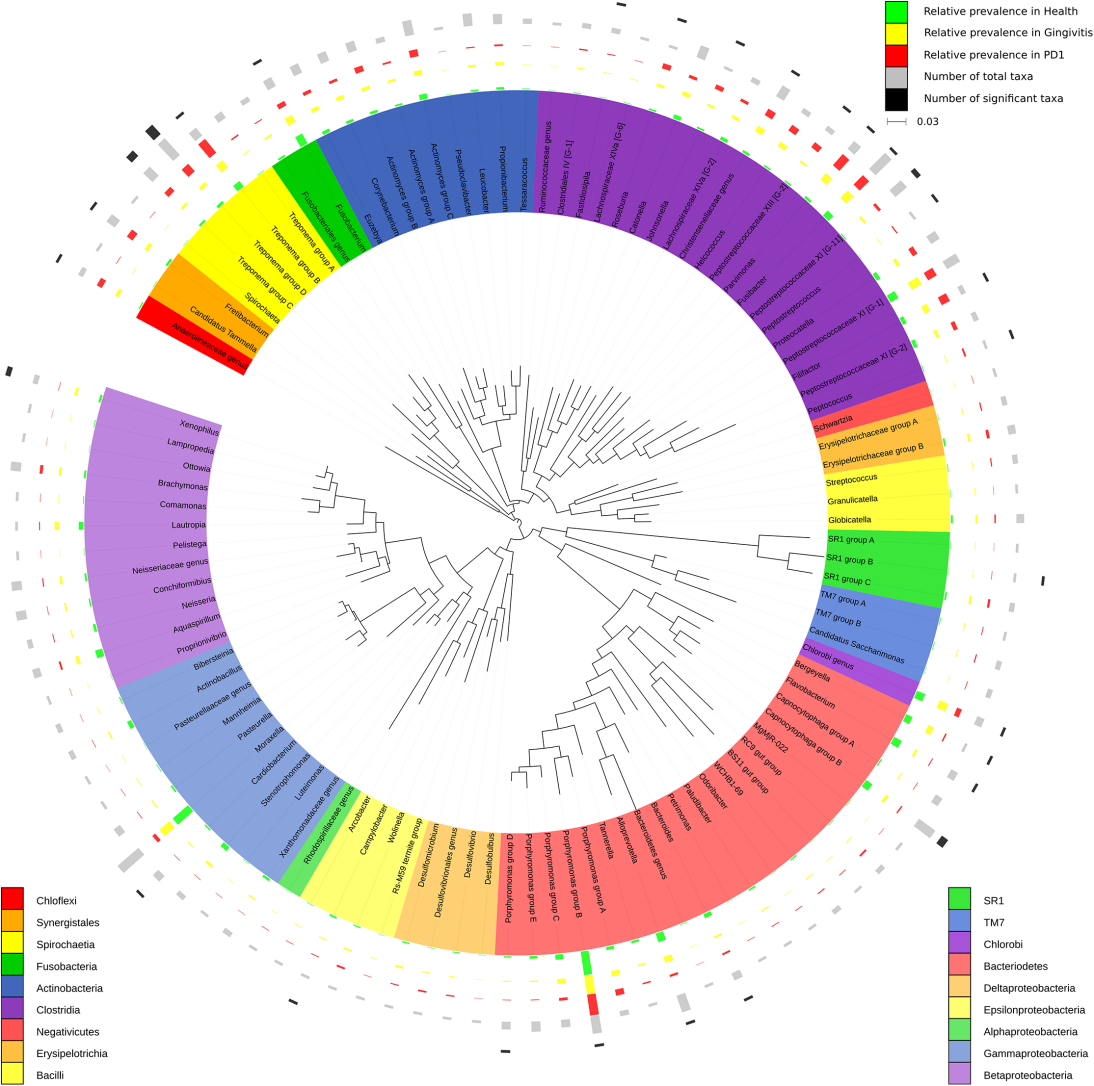
Circular maximum likelihood tree of full length 16S rRNA genes at the genus level. The inner band shows genera coloured by phylum/class (based on NCBI taxonomy). The next three bands depict relative abundance of each genus in health (green), gingivitis (yellow) and mild periodontitis (red). The two outer bands show the total number of taxa identified within the genus (grey, ranging from 1 to 11) and number of those taxa that showed a significant association with a health status (black, ranging from 0 to 5). A species level representation of the figure is given in S1 Fig.

A phylogenetic tree inferred at the genus level is shown in Fig 1 and at the species level in S1 Fig. The 75 genera observed contained 210 species of which the 34 most abundant species accounted for approximately 50% of the sequences (Fig 1, Table 1).

**Table 1:** The 34 most abundant OTUs in plaque from cats with healthy gingiva, gingivitis and mild periodontitis.

Species	Percentage identity	Total number of sequence reads	Proportion of total sequence reads (%)	Cumulative %
OTU8255 Peptostreptococcaceae XIII [G-1] bacterium FOT-028	100	32599	2.93	2.93
OTU7092 Porphyromonas sp. FOT-110	99.71	29691	2.64	5.57
OTU8837 Porphyromonas canoris	99.14	27727	2.41	7.98
OTU1062 Fusobacterium sp. FOT-120	99.43	24851	2.20	10.18
OTU3989 Treponema sp. FOT-201	100	22825	2.07	12.25
OTU1079 Peptostreptococcaceae XI [G-1] bacterium FOT-036	99.71	22462	2.10	14.35
OTU1473 Moraxella sp. FOT-087	100	22331	1.88	16.23
OTU7082 Bergeyella zoohelcum strain 357 FOT-329	99.71	21744	2.04	18.27
OTU2094 Chlorobi bacterium COT-312	98.86	19715	1.69	19.96
OTU906 Clostridiales [F-1][G-2] bacterium FOT-072	100	18494	1.78	21.73
OTU7952 Porphyromonas circumdentaria FOT-102	100	18292	1.57	23.30
OTU6282 Porphyromonas gulae FOT-105	99.71	17759	1.68	24.98
OTU7692 Moraxella sp. FOT-089	100	16027	1.40	26.38
OTU574 Filifactor villosus FOT-044	100	15704	1.45	27.83
OTU1976 Aquaspirillum sp. FOT-080	100	15400	1.32	29.15
OTU1500 Chlorobi [G] bacterium FOT-101	99.14	15304	1.39	30.54
OTU2476 Lautropia sp. COT-175	99.43	14565	1.29	31.83
OTU7091 Chloroflexi bacterium FOT-333	100	14227	1.33	33.16
OTU473 Helcococcus sp. COT-140	100	13695	1.24	34.40
OTU8729 Peptostreptococcaceae XI [G-4] bacterium FOT-065	100	13519	1.25	35.65
OTU4533 Actinomyces sp. FOT-320	100	13321	1.30	36.95
OTU7613 Lachnospiraceae XIVa [G-5] bacterium FOT-021	100	13107	1.21	38.16
OTU5191 Fretibacterium sp. FOT-215	100	12683	1.16	39.32
OTU6461 Capnocytophaga sp. FOT-330	100	11841	0.97	40.29
OTU8764 Treponema sp. COT-249	99.71	11443	1.07	41.36
OTU4910 Treponema sp. COT-207	99.71	11238	1.02	42.38
OTU7305 Lachnospiraceae XIVa [G-3] bacterium FOT-156	99.71	11197	1.07	43.45
OTU2615 Actinomyces sp. COT-404	100	11073	0.99	44.44
OTU6787 Porphyromonas sp. COT-290	99.14	10977	0.97	45.41
OTU9414 Lachnospiraceae XIVa [G-2] bacterium FOT-007	99.14	10697	0.96	46.37
OTU2425 Filifactor sp. FOT-129	99.43	10598	0.93	47.30
OTU298 Peptostreptococcaceae XI [G-1] bacterium FOT-035	100	10518	0.97	48.27
OTU2679 Proteocatella sp. FOT-127	100	10459	0.95	49.22
OTU883 Peptostreptococcaceae XI [G-1] bacterium FOT-040	100	9958	0.90	50.12

When considering abundance across all three disease states no one species was especially dominant, with the most abundant species *Peptostreptococcaceae XIII [G-1] bacterium FOT-028* (OUT 8255) averaging 2.93% of sequence reads. A number of other species had very similar abundance levels to this, with six other species each accounting for between 2% and 2.7% of reads (*Treponema sp*. *FOT-201* (OTU 3989), *Moraxella sp*. *FOT-087* (OTU 1473), *Clostridiales [F-1][G-2] bacterium FOT-072* (OTU 906), *Porphyromonas circumdentaria FOT-102* (OTU 7952), *Moraxella sp*. *FOT-089* (OTU 7694) and *Filifactor villosus FOT-044* (OTU 574). Many of these species were equally prevalent in health, gingivitis and mild periodontitis and were not associated with a disease state (as discussed below). A further 21 OTUs represented between 1% and 2% of the population and the remaining 239 OTUs ranged in abundance from 0.01% and 0.99%.

### The core microbiota

**Fig 2:**
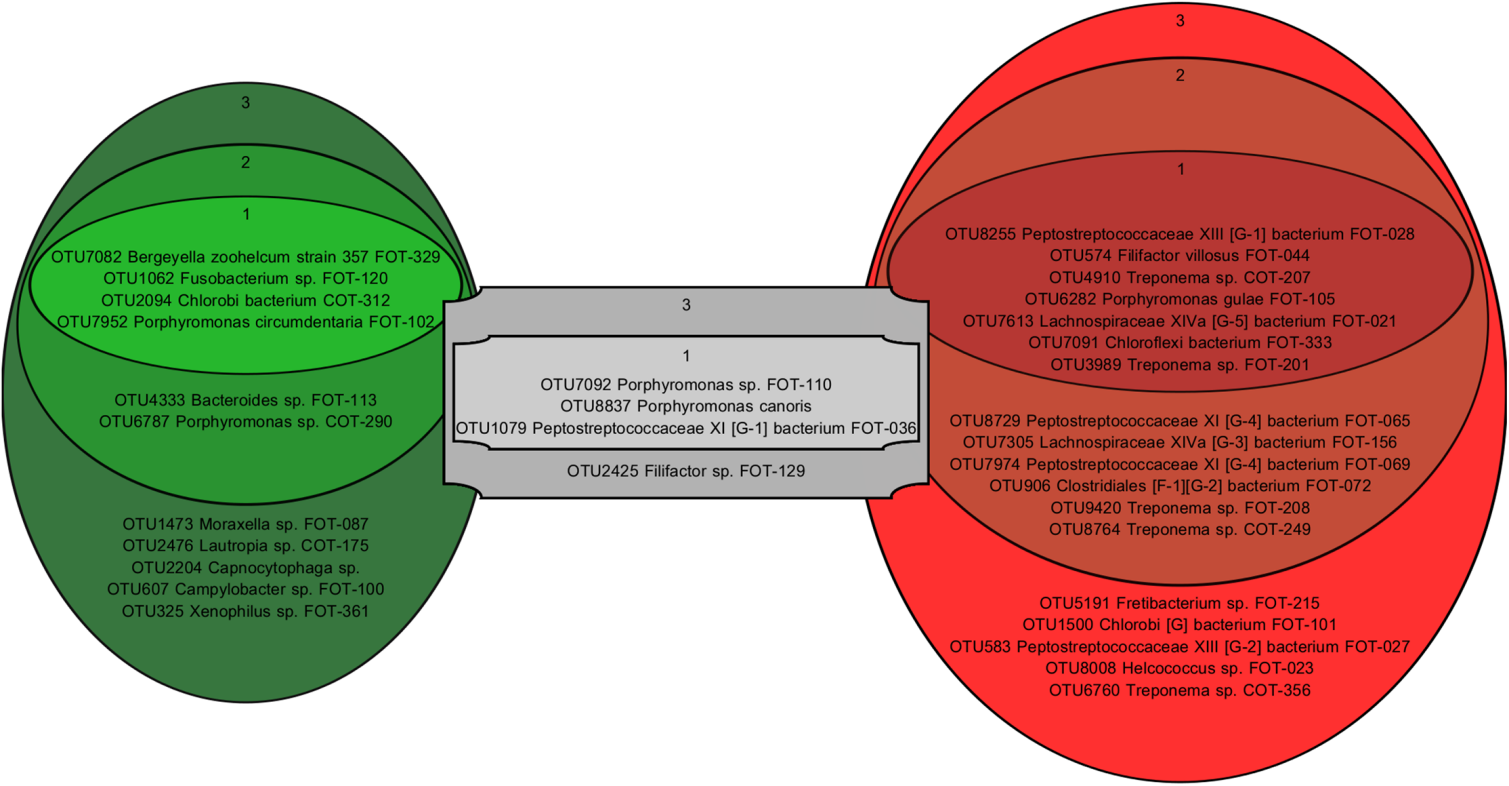
The feline construal of the core microbiota in health and mild periodontitis figure from Abusleme *et al*. (2013). OTUs were defined as part of the core microbiota if they represented ≥0.5% of sequence reads in at least 50% of samples. If they were equally prevalent and abundant in healthy and mild periodontitis samples they formed part of the core microbiota (grey rectangles). If they were more prevalent and abundant in healthy samples they formed part of the healthy core (green ovals). If they were more prevalent and abundant in mild periodontitis samples they formed part of the mild periodontitis core microbiota (red ovals). Relative prevalence and abundance data were used for further sub-division. Ovals labelled with 1 contain highly prevalent (≥2/3^rd^ of samples) and highly abundant OTUs (≥2% of sequences). Ovals labelled with 2 contain highly prevalent (≥2/3^rd^ of samples) with lower abundances (<2% of sequences). Ovals labelled with 3 contain less but still highly prevalent OTUs (≥1/2 and <2/3^rd^ of samples).

The prevalence and abundance of common OTUs was analysed to identify if there were core microbiota. For the purposes of this description, prevalence is defined as the proportion of samples that contain a particular OTU and the abundance is the proportion of sequences that an OTU comprises within a sample or population. Some OTUs were associated primarily with healthy samples, others with samples from mild periodontitis, whilst some were prevalent in all samples regardless of health state (Fig 2). The core species were defined as those present at ≥0.5% abundance in the majority of samples (50% or more) with similar prevalence between health and disease (Fig 2, grey rectangles). The most prevalent members of this group were two *Porpyhromonas* species, (*P*. *canoris* [25] and an uncharacterised feline *Porpyhromonas* species (FOT-110)) along with *Peptostreptococcaceae XI [G-1] bacterium* FOT-036. One other species, *Filifactor* sp. FOT-129 was less prevalent but still present in the majority of samples with equal prevalence and relative abundance in health and disease (Fig 2). The healthy core species were defined as those present in the majority of health samples at ≥0.5% abundance and found at an increased prevalence and abundance in health samples compared to mild periodontitis (Fig 2, green rectangles). Within this group were 5 abundant species (*Moraxella sp*. *FOT-087*, *Bergeyella zoohelcum strain 357 FOT-329*, *Fusobacterium sp*. *FOT-120*, *Chlorobi bacterium COT-312* and *Porphyromonas circumdentaria FOT-102*). A further 2 species (*Porphyromonas sp*. *COT-290* and *Bacteroides* sp. FOT-113) were less abundant but highly prevalent with a further four other species less abundant and less prevalent but still present in the majority of health samples. In contrast, there was a greater conservation of species in the mild periodontitis samples with a core of 7 highly prevalent and highly abundant species and a further 7 less abundant but still highly prevalent species (Fig 2, red rectangles). A further 4 species were less abundant and less prevalent but still present in the majority of samples. The 19 core disease species were dominated by members of the class Clostridia (9) and the genus *Treponema* (6).

### Associations between health and disease

**Fig 3:**
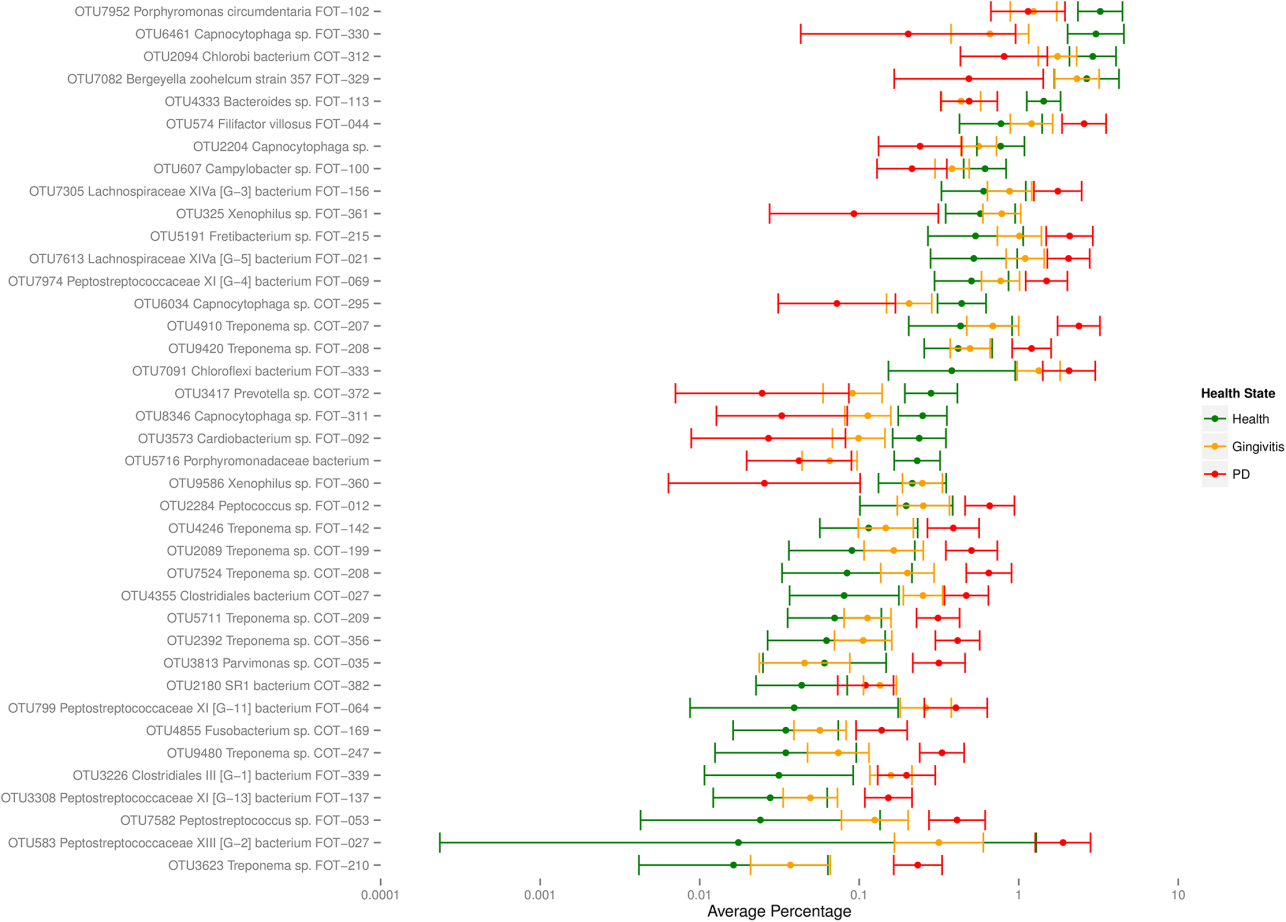
Average percentage prevalence with 95% confidence intervals for OTUs with significant health status effect in; health (green), gingivitis (yellow) and mild periodontitis (red).

Health and disease associations were determined by logistic regression analysis. Of the 267 OTUs, 39 showed a statistically significant health status effect after a permutation test for distributional assumptions, followed by multiplicity correction. Of these, 16 showed a statistically significant difference between health and gingivitis, 30 showed a statistically significant difference between gingivitis and mild periodontitis and all 39 showed a statistically significant difference between health and mild periodontitis (Fig 3, S4 Table). Of the most abundant health associated species (those >1% average abundance), only *Chlorobi bacterium COT-312* (OTU# 2094 2.74% in health) showed statistically significant differences in abundance between all three health states. Three other species, *Porphyromonas circumdentaria FOT-102* (OTU# 7952 3.03%), *Capnocytophaga sp*. *FOT-330* (OTU# 6461 2.7%) and *Bacteroides sp*. *FOT-113 (OTU# 4333 1*.*39%)* were significantly more abundant in health than in either gingivitis or mild periodontitis but were not significantly different between gingivitis and mild periodontitis. In addition one other abundant health species *Bergeyella zoohelcum strain 357 FOT-329* (OTU# 7082 2.04%) was significantly more abundant in health samples than in mild periodontitis samples and also significantly more abundant in gingivitis samples than in mild periodontitis but did not show a significant difference between health and gingivitis samples (S4 Table).

The two most abundant species to be statistically significantly associated with mild periodontitis *were Filifactor villosus* FOT-044 (OTU# 574 average 2.5% in mild periodontitis) and *Treponema sp*. *COT-207* (OTU# 4910 2.3%). These species showed statistically significant differences between mild periodontitis and health and between mild periodontitis and gingivitis but not between health and gingivitis (Fig 3, S4 Table). Five other abundant species (average >1%) also followed the same pattern; *Fretibacterium sp*. *FOT-215* (OTU# 5191 2.2%), *Lachnospiraceae XIVa [G-3] bacterium FOT-156* (OTU# 7305 1.8%), *Peptostreptococcaceae XIII [G-2] bacterium FOT-027* (OTU# 583 1.72%), *Peptostreptococcaceae XI [G-4] bacterium FOT-069* (OTU# 7974 1.49%), *Treponema sp*. *FOT-208* (OTU# 9420 1.21%). Of the other abundant disease associated species, *Lachnospiraceae XIVa [G-5] bacterium FOT-021* (OTU# 7613 2.1%) showed statistically significant differences in abundance between all three health states and *Chloroflexi bacterium FOT-333* (OTU# 7091 2.03%) was significantly more abundant in mild periodontitis than either health or gingivitis but did not show a significant difference between health and gingivitis (Fig 3, S4 Table).

**Fig 4:**
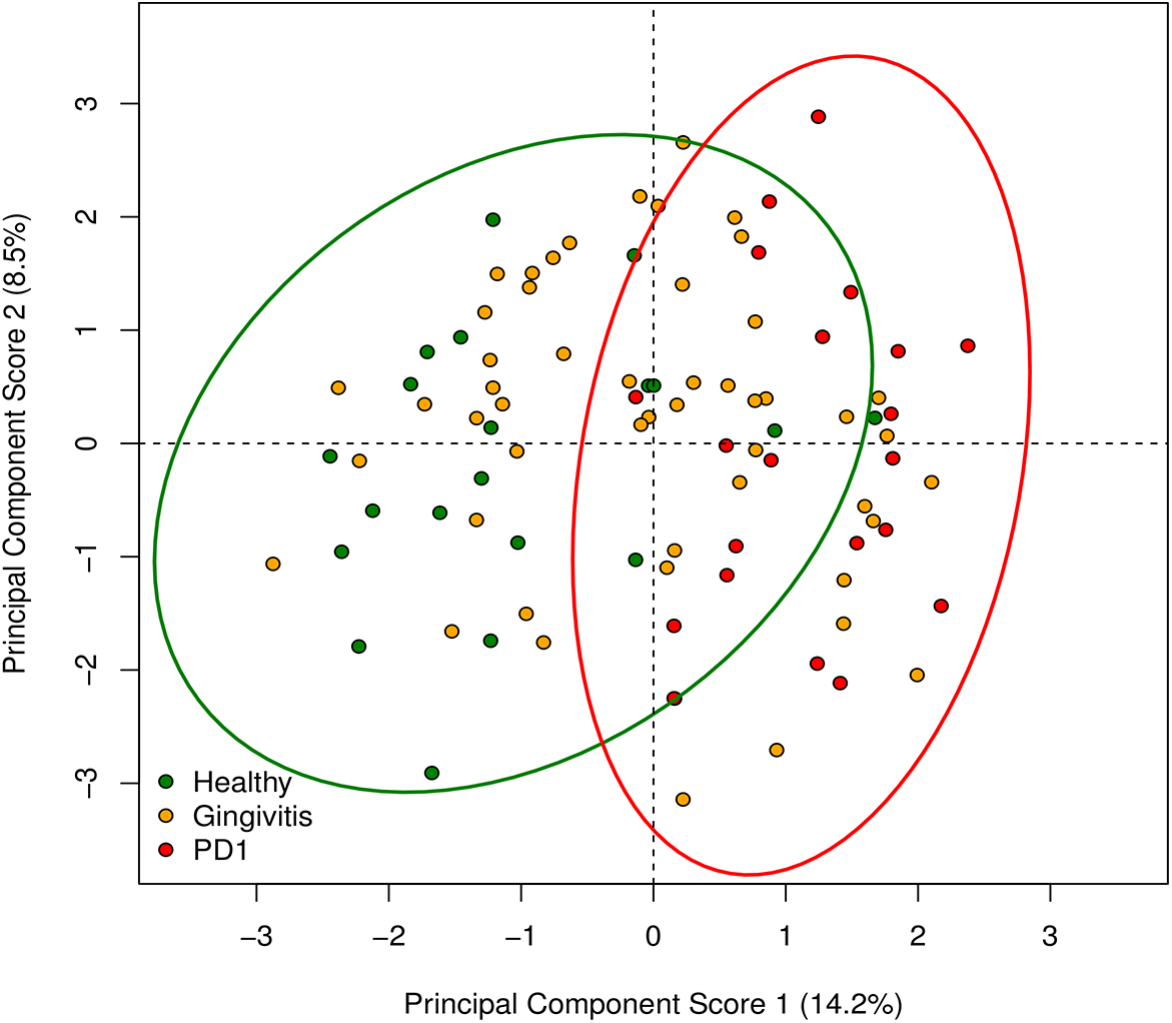
PCA scores from analysis performed on the log_10_ proportions of OTUs identified in each individual coloured by health status; health (green), gingivitis (yellow) and mild periodontitis (red). The 95% confidence ellipses for the scores were calculated by the R package vegan.

Principal component analysis (PCA) was used to investigate correlations between OTUs and health state, age and gender. The first component explained 14% and the second component 8.78% of the variability in the OTUs (Fig 4). Discrete clustering of health and mild periodontitis samples was observed whilst gingivitis samples overlaid both the health and mild periodontitis clusters. Age and gender did not show any discrete clusters (data not shown).

**Fig 5:**
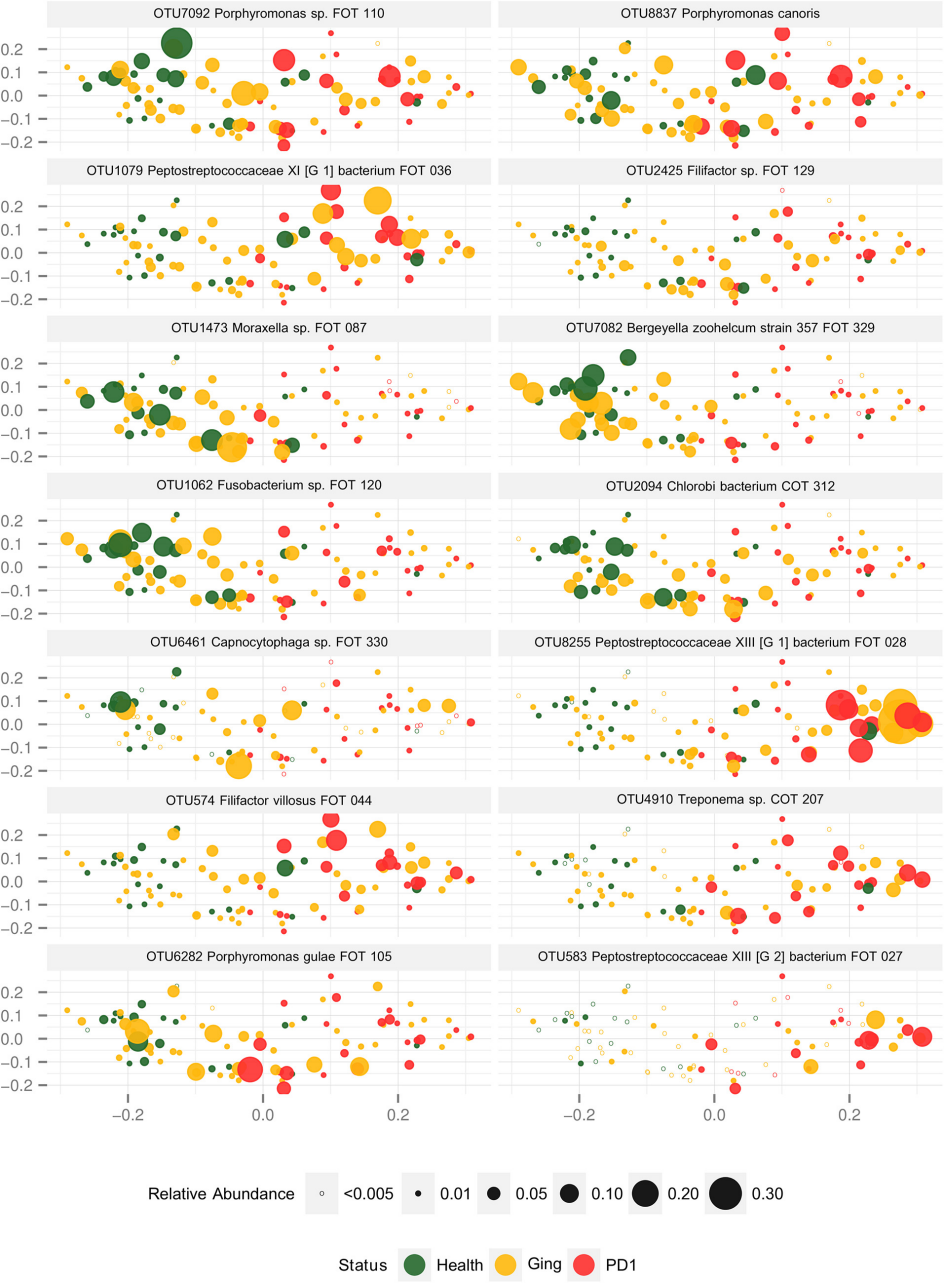
PCoA plots of the JSD distance for samples by disease status. All plots show the ordination of all samples according to the PCoA analysis. Each panel depicts a different OTU. Each point represents a sample and the area of the point is weighted according to the relative abundance of the OTU being depicted in the panel. Hollow circles indicate OTU’s with zero counts or relative abundances less than 0.5% per sample. The colour of the point indicates whether the sample was from health (green), gingivitis (orange) or mild periodontitis (red).

The abundance of selected species within individual samples was visualised by Principal coordinates analysis (PCoA) using JSD distances. Fig 5 shows a series of PCoA plots where each data point is weighted according to the abundance of the OTU being depicted in the plot in question. Selected members of the core species, core health species and core periodontitis species (Fig 2) are shown.

### Association with average gingivitis score

**Fig 6:**
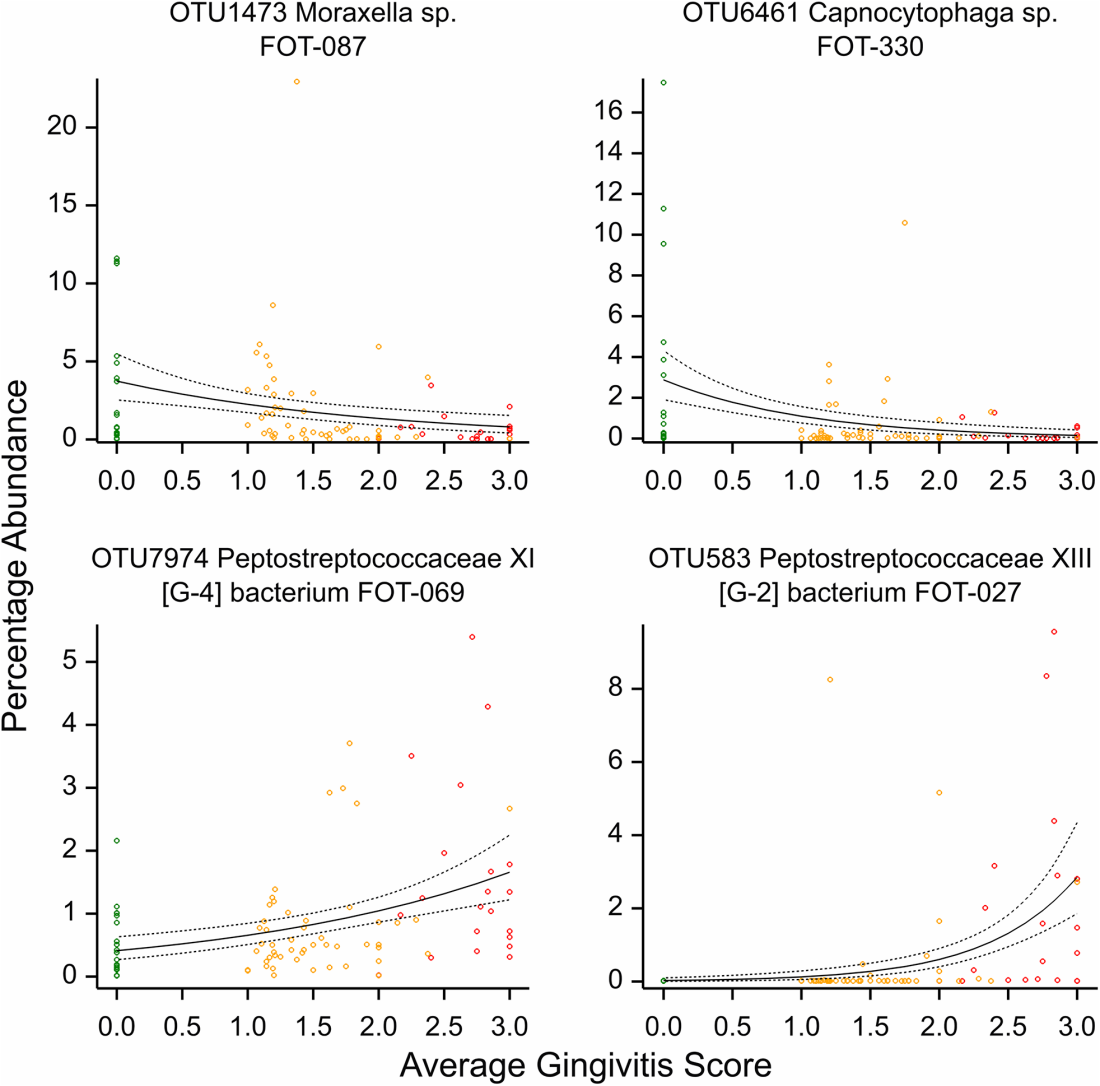
Percentage abundance for four different OTUs, plotted against the average gingivitis score of the sampled teeth, with 95% confidence limits, including the raw data coloured by health status; health (green), gingivitis (yellow) and mild periodontitis (red).

Average gingivitis scores were collected for each sample allowing a comparison between gingivitis score and the abundance of each individual OTU to be made to look for OTUs that vary across the gingivitis scale. Of the 267 OTUs (plus rare), 58 were found to have a statistically significant correlation with the average gingivitis score of the sampled teeth (after a permutation test for distributional assumptions, and followed by a multiplicity correction) (S5 Table). Twenty three of these OTUs decreased significantly from an average gingivitis score of 0 to a score of 3, the other 35 increased significantly. The majority of the OTUs that decreased significantly as the gingivitis score rose followed a similar pattern, as exemplified by *Moraxella sp*. *FOT-087* (Fig 6). The exception to this was *Capnocytophaga sp*. *FOT-330* (Fig 6) which showed a steeper curve. The same was true for the OTUs that increased as the gingivitis score rose. Again the majority showed a similar shape of curve exemplified by *Peptostreptococcaceae XI [G-4] bacterium FOT-069* (Fig 6). The exceptions to this were *Treponema sp*. *COT-207* and in particular *Peptostreptococcaceae XIII [G-2] bacterium FOT-027* (Fig 6) which both rose much more steeply as the gingivitis score increased.

### Gram-stain status and oxygen requirements

**Fig 7:**
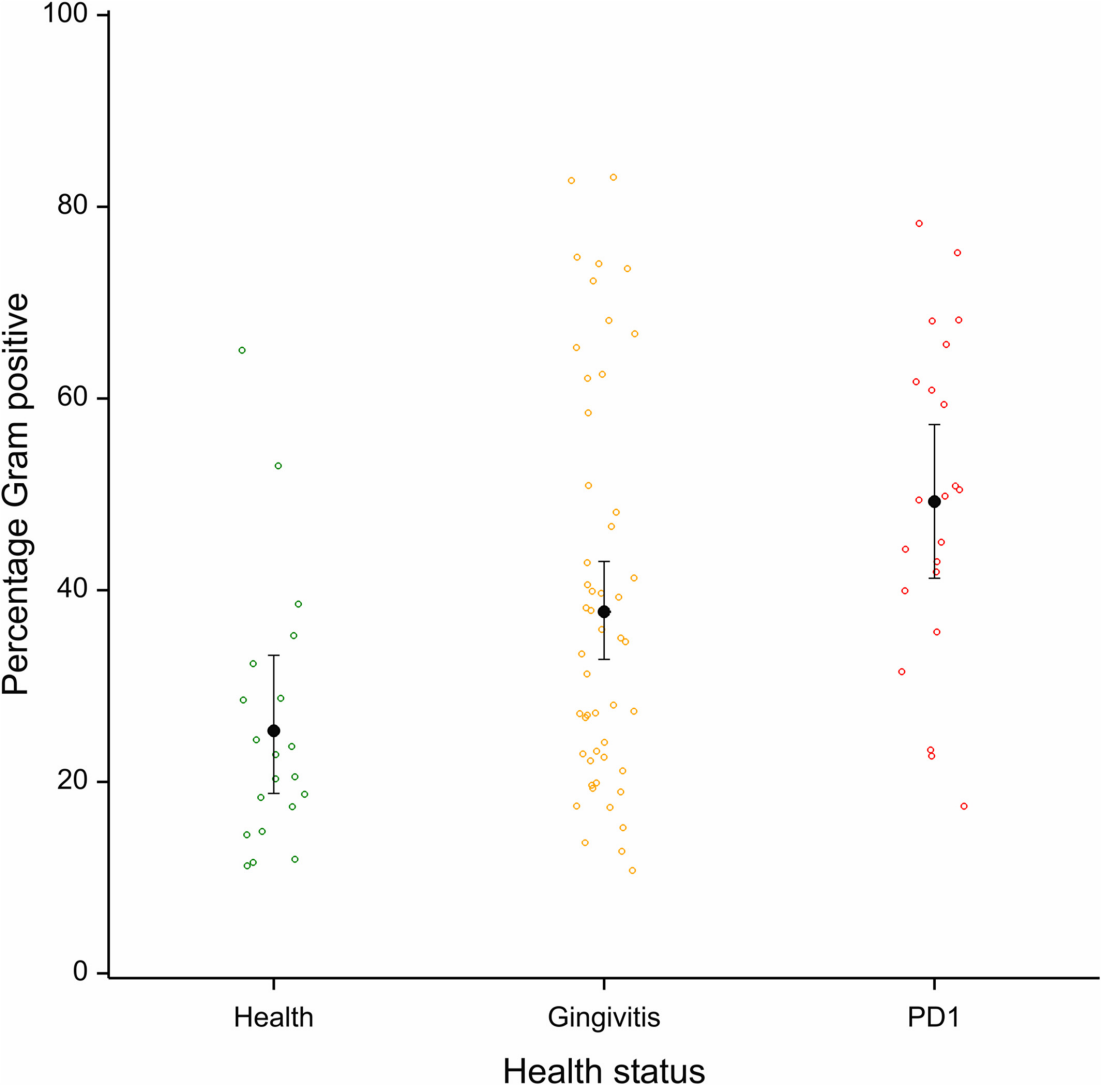
Percentage abundance of Gram positive OTUs for each sample by health status; health (green), gingivitis (yellow), mild periodontitis (red). Black bars indicate mean percentage of OTUs that are Gram positive with 95% confidence intervals.

The proportion of Gram positive and Gram negative non-rare OTUs is shown in Fig 7 (as well as S2 Fig and S6 Table). The Gram status profile was significantly different in health, gingivitis and mild periodontitis. Samples from cats with mild periodontitis had a significantly higher proportion of Gram positive species than cats with gingivitis (*p* = 0.019) or healthy gingiva (*p*<0.001). Gingivitis samples also had a significantly higher proportion of Gram positive species than the healthy samples (*p* = 0.01).

**Fig 8:**
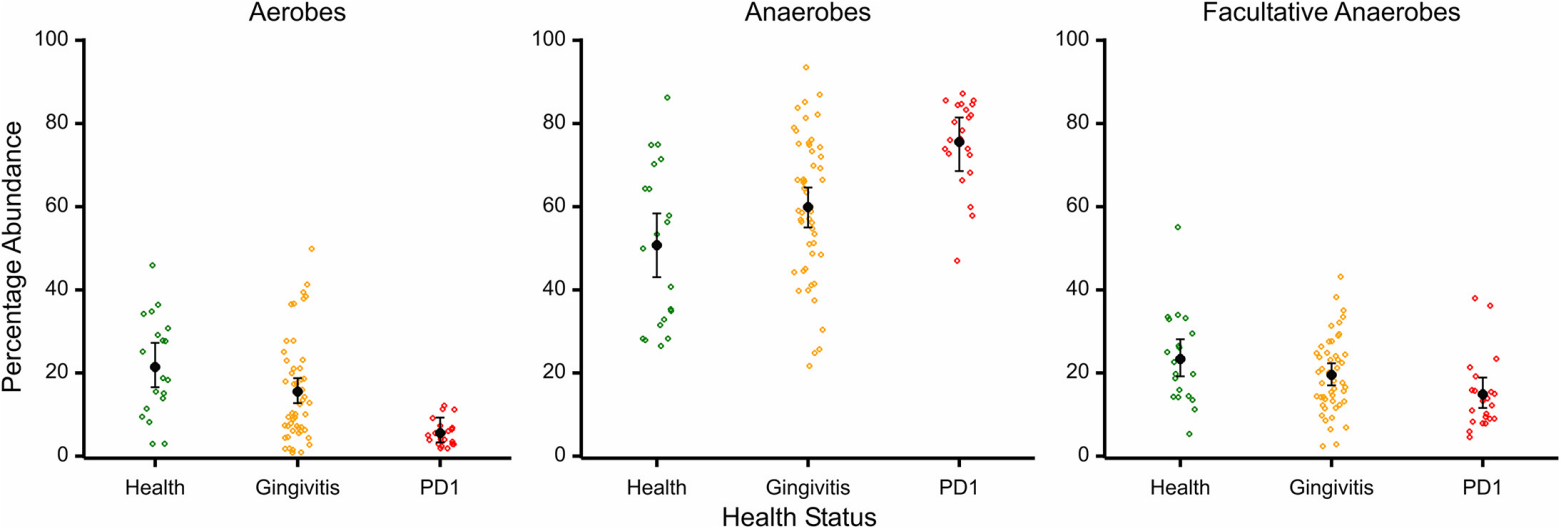
Percentage abundance of A) aerobes, B) anaerobes and C) facultative anaerobes for each sample by health status; health (green), gingivitis (yellow), mild periodontitis (red). Black bars indicate mean percentage of OTUs that are aerobic, anaerobic or facultatively anaerobic with 95% confidence intervals.

The proportions of aerobes, anaerobes and facultative anaerobes are shown in Fig 8 and S7 Table. Statistically significant differences in oxygen requirements were observed between the bacterial populations in health, gingivitis and mild periodontitis samples. Health samples contained a significantly higher proportion of aerobes than either gingivitis samples (*p* = 0.047) or mild periodontitis samples (*p*<0.001) and gingivitis samples contained a higher proportion than mild periodontitis samples (*p*<0.001). Mild periodontitis samples contained a significantly higher proportion of anaerobes than either gingivitis samples (*p*<0.001) or health samples (*p*<0.001). Likewise gingivitis samples contained a higher proportion of anaerobes than health samples (*p* = 0.048). Finally facultative anaerobes were only significantly different between the health and mild periodontitis samples (*p* = 0.005).

### Species richness and diversity

A linear model was used to compare the number of OTUs, including rare OTUs, in health, gingivitis and periodontitis. The number of OTUs was not found to be significantly different between any of the health states (overall effect *p* = 0.175) (S8 Table & S3 Fig). A linear model was also used to analyse diversity using the Shannon index. The Shannon diversity was not found to be significantly different between any of the health states (overall effect *p* = 0.255) (S9 Table & S4 Fig).

## Discussion

This is the first study of the feline oral microbiota that has been performed in sufficient depth and with sufficient samples to be able to represent the diversity of bacterial species that are found in cats’ mouths. Our previous studies [9, 10], which provided the full length 16S rRNA sequences from 171 feline oral taxa and 282 canine oral taxa respectively, were crucial to allow us to extract meaningful information from the relatively short reads provided by the 454 technology. The indication is that the majority of the most prevalent species have now been found (within the limits of the method used, which relies on universal PCR primers) since 85% of the OTUs identified mapped to within 98% identity to known species in FOMD, COMD or Silva. In fact greater than 90% of the sequences mapped at the species level ≥98% identity (S3 Table) to the combined database and 16 of the top 20 OTUs mapped to species in the FOT database (Table 1) underlining the value of Dewhirst *et al*.*’s* previous cat study [9]. When assigning which species an OTU mapped to some difficulties were encountered. Due to the short sequence length, in some cases the OTU mapped to multiple different species in the combined database at >98% identity. Often there were both FOT and COT OTUs that mapped at similar levels of identity. In general we assigned the OTU to a FOT if greater than 99% identity even if a more similar hit existed in COMD or Silva on the basis that with the length of the 454 reads we were only sampling a small portion of the 16S gene.

We applied a cut off of 0.05% for rare sequences based on mock community data which indicated that below this level 454 sequencing was not able to distinguish rare sequences from sequencing artefacts (Davis et al., 2013). This resulted in the identification of 267 OTUs plus a “rare” group. In contrast to our dog study [10] and previous human studies [26, 27] we did not see any differences in species diversity or in OTU number between the different health states sampled. We did however observe differences between the proportions of aerobes, anaerobes and facultative anaerobes between the different health states. Anaerobic species dominated in all health states making up 50% of species in health and nearly 80% in mild periodontitis samples, as we previously observed in dog plaque. Particularly striking was the low abundance of aerobic species in plaque from mild periodontitis, which varied from 2% to just over 10% (Fig 8). This association between anaerobic species and periodontitis is well documented, the supposition being that the anoxic environment of periodontal pockets selects for anaerobic species [28].

The proportion of Gram positive species was higher in mild periodontitis than it was in health as was observed previously in the dog study and in contrast to some human studies. In fact the cat data mirror to a remarkable extent what was found in the dogs. Healthy cat plaque is dominated by Gram negative species (approx. 75%) and plaque from mild periodontitis contains an equal proportion of Gram positive species and Gram negative species. This difference is caused by a reduction in Proteobacteria and Bacteriodetes species and an increase in Firmicutes, which double in proportion from approx. 20% of the population in health to approx. 40% of the population in mild periodontitis (Table 2). When the oral bacteria are examined at the phylum level an interesting observation emerges. Five phyla (Firmicutes, Spirochetes, Syngeristetes, Chloroflexi and TM7) that are increased in feline periodontitis relative to health are also increased in human periodontitis [26]. The differences between cat and human therefore appear to be mostly in the phyla found in healthy plaque, which mirrors what we found previously for dog versus human [29]. The major difference between the data shown here and that reported in most human studies is the reduced proportion of Actinobacteria in feline healthy plaque. In our study, Actinobacteria were a minor player at <10% in all health states. In contrast, human studies indicate that in healthy plaque Actinobacteria can make up over 50% of the bacteria [26, 27].

**Table 2:** The percentage abundance of phylum in plaque from cats with healthy gingiva, gingivitis and mild periodontitis.

Phylum	Health	Gingivitis	PD1
Bacteroidetes	29.61	20.21	16.23
Proteobacteria	24.04	17.54	6.72
Firmicutes	19.11	30.57	40.47
Actinobacteria	7.29	8.78	8.48
Fusobacteria	5.42	3.59	2.05
Spirochaetae	4.32	5.98	13.46
Chlorobi	3.22	3.60	1.91
Synergistetes	0.83	1.59	2.68
Candidate_division_SR1	0.74	1.03	0.92
Candidate_division_TM7	0.35	0.63	0.70
Chloroflexi	0.35	1.42	2.13

With the above differences in mind it is not too surprising that the core microbiome in health identified here (Fig 2) does not overlap at all with that found for healthy human plaque [26]. In fact, the core microbiota found in healthy cat plaque are much more similar to the common species in healthy dog plaque. Of the feline core healthy species, *Porphyromonas circumdentaria FOT-102*, *Bergeyella zoohelcum strain 357 FOT-329*, *Moraxella sp*. *FOT-087*, *Capnocytophaga sp*. and *Lautropia sp*. *COT-175* all have health associated equivalent species in dog plaque (*Porphyromonas cangingivalis*, *Bergeyella zoohelcum*, *Moraxella COT-396*, *Capnocytophaga sp*. *COT-339*, *Lautropia sp*. *COT-175* [29]). In terms of disease associated bacteria, cat and dog also share many commonalities. The feline mild periodontitis core species include several *Peptostreptococcaceae*, *Filifactor villosus*, a member of the *Lachnospiraceae XIVa [G-5] genus*, *Helcoccus and Clostridiales* species, all of which have disease associated relatives in canine plaque. The most prevalent disease associated species of all *Peptostreptococcaceae XIII [G-1] bacterium FOT-028* is 100% identical over the region sequenced to *Peptostreptococcaceae bacterium COT-030* which is the 4^th^ most abundant canine Peptostreptococcaceae species and also disease associated in the dog [29]. In addition, within the core species that are common to all health states, *Porphyromonas canoris* has a similar distribution in dog and cat and *Porphyromonas sp*. *FOT-110*, although not closely related, has a similar pattern of prevalence to *Porphyromonas gingivicanis* [29]. In addition to many similarities, there were also a few differences between cat and dog. Feline plaque contained a *Choroflexi* and a *Chlorobi* species both of which were disease associated and reasonably abundant in cats but rare in dogs. Also, the plaque from mild feline periodontitis contained a much larger number of disease associated *Treponeme* species than was observed in dog plaque (Fig 3, [29]).

Comparing the bacterial species found in plaque from different gingival health states in different host species allows common themes to be identified. This has the potential to refine our knowledge of which species are important in disease progression, on the basis that species that are associated with a particular health state in more than one host species are more likely to be important for that health state. The bacterial species present in feline and canine healthy plaque are similar to one another. In contrast the species found in human plaque, especially healthy human plaque are very different. This could be as a result of a western diet that is high in sugar leading to a proliferation of Streptococcal species along with species that depend on them such as Veillonella. This idea is supported by a recent human study [30] that showed a much lower abundance of streptococcal species (~7%) and Actinobacteria in general (~15%) in human subjects from a rural part of China who consume a diet naturally low in sugar (Wenyuan Shi personal communication). As such, human oral bacterial associations are more complex to interpret because of the potentially competing interactions between the bacteria responsible for two different disease processes (i.e. periodontitis and dental caries). This suggests that studying the bacterial associations in host species that have very low levels of dental caries such as cats and dogs may facilitate progress towards understanding the cause of periodontitis.

Very few species are good candidates for being diagnostic of either health or disease in our data. Although a PCA analysis was able to separate the health from the mild periodontitis samples, with the gingivitis samples over laying both groups (Fig 4), we found tremendous individual variation between samples and even the core species were not evenly distributed (Fig 5). No species were confined to just health or disease and in fact species that were health or disease associated on the whole still reached high levels in the gingivitis samples and occasionally even in the opposite health state (for example OTU574 *Filifactor villosus* FOT044). As previously hypothesised, the changes that occurred as periodontitis developed involved shifts in the proportions of species that were already there rather than new species arriving or being replaced [26].

One species does appear to be a good marker for mild periodontitis if it is present. *Peptostreptococcaceae XIII [G-2] bacterium FOT-027* reaches high proportions in many disease samples and no health samples (Fig 5) although it is not a universal feature of mild periodontitis samples being present in only 50% of them. Interestingly a very close homologue in canine plaque was also diagnostic of approaching periodontitis in a recent study [31]. Out of 35 species associated with increasing gingivitis score *Peptostreptococcaceae XIII [G-2] bacterium FOT-027* was one of only two with a different pattern (the other being *Treponema sp*. *COT-207*) with a steeper curve with advancing gingivitis scores. It is therefore difficult to tell whether this species is a marker for advanced gingivitis or mild periodontitis and also whether it is responding to events or orchestrating them.

Another potentially relevant species is *Porphyromonas gulae*. The feline *P*. *gulae* strain seems to have a very similar profile to the canine one being present at an average abundance of about 1.1% in health and 2.2% in mild periodontitis samples. *P*. *gulae* is very closely related to the keystone human oral pathogen *P*. *gingivalis*. In humans *P*. *gingivalis* has been studied extensively and is strongly associated with periodontitis and rarely found in health. In contrast, in cats and dogs *P*. *gulae* is only marginally more abundant in mild periodontitis than in health. This is despite canine *P*. *gulae* strains having the vast majority of the known *P*. *gingivalis* genes [32]. *P*. *gulae* was also only marginally more prevalent in mild periodontitis samples (present 68% of the time) compared to 56% of the time in gingivitis and 45% of the time in healthy samples and showed no statistical association to any health state. Finally it reached high levels in many healthy and gingivitis samples (Fig 5). Without the wealth of research into *P*. *gingivalis* and its role in periodontitis it is difficult to imagine that *P*. *gulae* would be selected as especially meriting further study for its potential role in feline periodontitis based on its prevalence and abundance profile.

One of the main differences from *P*. *gingivalis* is the reasonably high prevalence of *P*. *gulae* in healthy plaque. It is possible that this is masking the importance of *P*. *gulae* in disease. As a genus the *Porphyromonas* are more prevalent in general in both cats and dogs than in humans. One thing that cats and dogs share is a high salivary pH ([33]. It maybe, that the higher pH of cat and dog saliva favours the growth of porphyromonads either directly or indirectly by slowing the growth of competing species. Another intriguing possibility is that the difference relates to the fact that almost all animal *Porphyromonas* species contain a catalase gene that human Porphyromonad species lack [32]. The presence of this gene may help to support growth under aerobic conditions by metabolising hydrogen peroxide allowing the porphyromonads to survive more successfully in healthy plaque.

We have performed the first comprehensive study on the bacteria associated with feline health and mild periodontitis. The bacteria identified share a lot in common with bacteria found in canine plaque, but differ extensively from those in the human oral microbiome. This is perhaps not surprising given similarities in canine and feline diets and some oral parameters such as salivary pH. It implies that interventions targeted at human pathogenic species will not be effective for use in cats but that there is more potential for commonalities in interventions for cats and dogs.

## Supporting Information

S1 Fig Circular maximum likelihood tree of full length 16S rRNA genes at the species level. The inner band shows species coloured by phylum/class (based on NCBI taxonomy). The next three bands depict relative abundance of each species in health (green, gingivitis (yellow) and mild periodontitis (red). The outer band (black) indicates whether species show a statistically significant association with one of the three health states.
(PDF)

S2 Fig Percentage abundance of Gram negative OTUs for each sample by health status; health (green), gingivitis (orange), mild periodontitis (red). Black bars indicate mean percentage of OTUs that are Gram negative with 95% confidence intervals.
(PDF)

S3 Fig Number of OTUs for each sample by health state; health (green), gingivitis (orange), mild periodontitis (red). Black bars indicate mean number of OTUs with 95% confidence intervals.
(PDF)

S4 Fig Shannon diversity index values for each sample by health state; health (green), gingivitis (orange), mild periodontitis (red). Black bars indicate mean Shannon diversity index for OTUs in that health state with 95% confidence intervals.
(PDF)

S1 Table Table listing Taxa grouped together for Genus level Plot. (XLSX)

S2 Table Metadata for all feline plaque samples used in this study. (XLSX)

S3 Table Abundance and homology data for all 267 OTUs. (XLSX)

S4 Table Data showing statistical associations between OTUs and health state. (XLSX)

S5 Table Data showing statistical associations between OTUs and average gingivitis score. (XLSX)

S6 Table Data for average abundance of Gram stain status (Table A) by health state and (Table B) by health status contrast odds ratios. (XLSX)

S7 Table Data for average abundance of aerobes/anaerobes/facultative anaerobes (Table A) by health state and (Table B) by health status contrast odds ratios. (XLSX)

S8 Table Number of OTUs (Table A) by health state and (Table B) by related health state contrast odds ratios. (XLSX)

S9 Table Shannon diversity index (Table A) by health state and(Table B) by related health state contrast odds ratios. (XLSX)

S10 Table Top 20 most similar species in FOMD/COMD/Silva for each out. (XLSX)
