# Supplementary figures and images for: A Pyrosequencing Investigation of Differences in the Feline Subgingival Microbiota in Health, Gingivitis and Mild Periodontitis
Source: PLoS One. 2015 Nov 25;10(11):e0136986. doi: 10.1371/journal.pone.0136986 (PMC4659563; doi:10.1371/journal.pone.0136986)

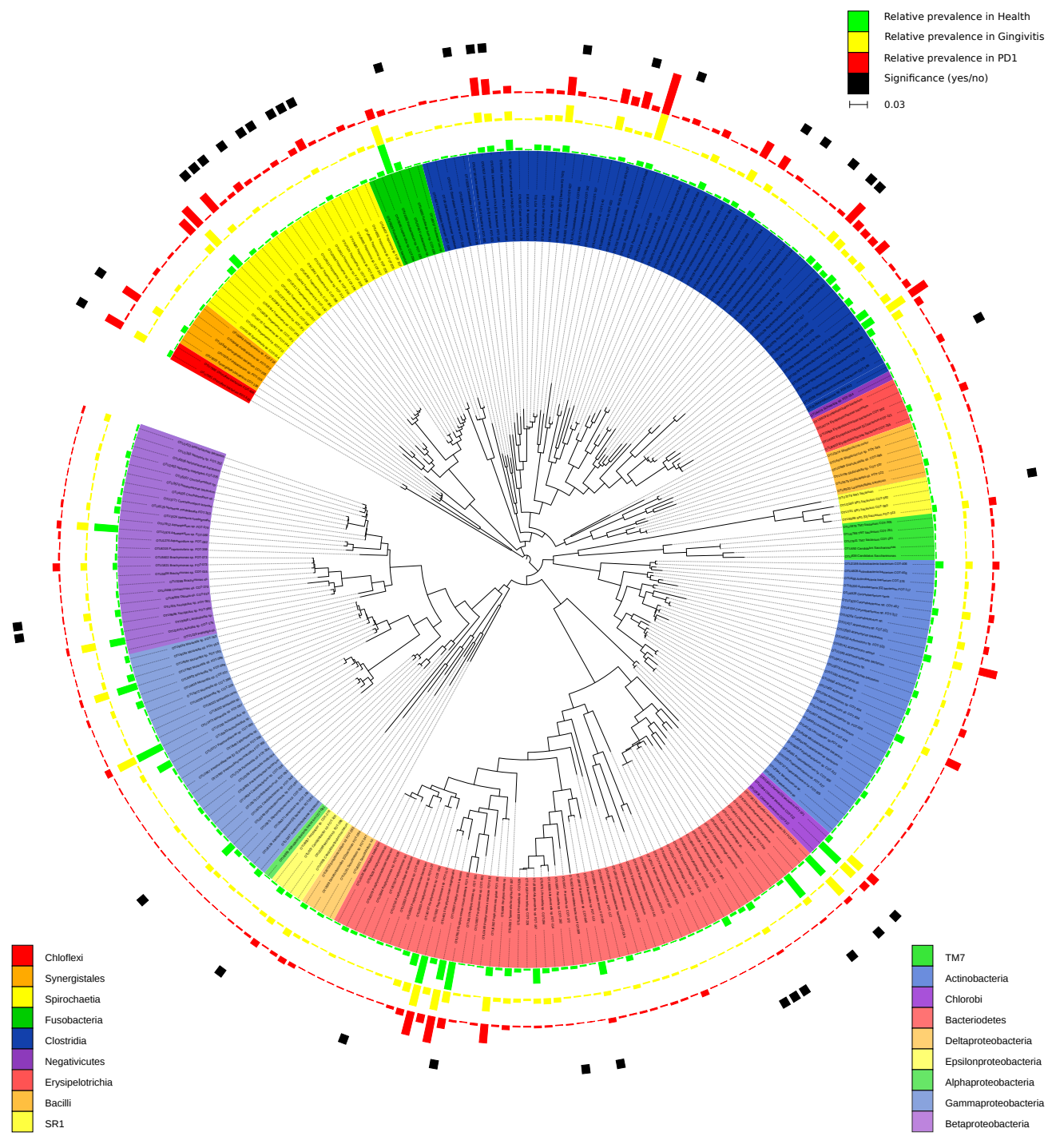

Supplement: S1 Fig — The inner band shows species coloured by phylum/class (based on NCBI taxonomy). The next three bands depict relative abundance of each species in health (green, gingivitis (yellow) and mild periodontitis (red). The outer band (black) indicates whether species show a statistically significant association with one of the three health states. (PDF) [file pone.0136986.s001.pdf]

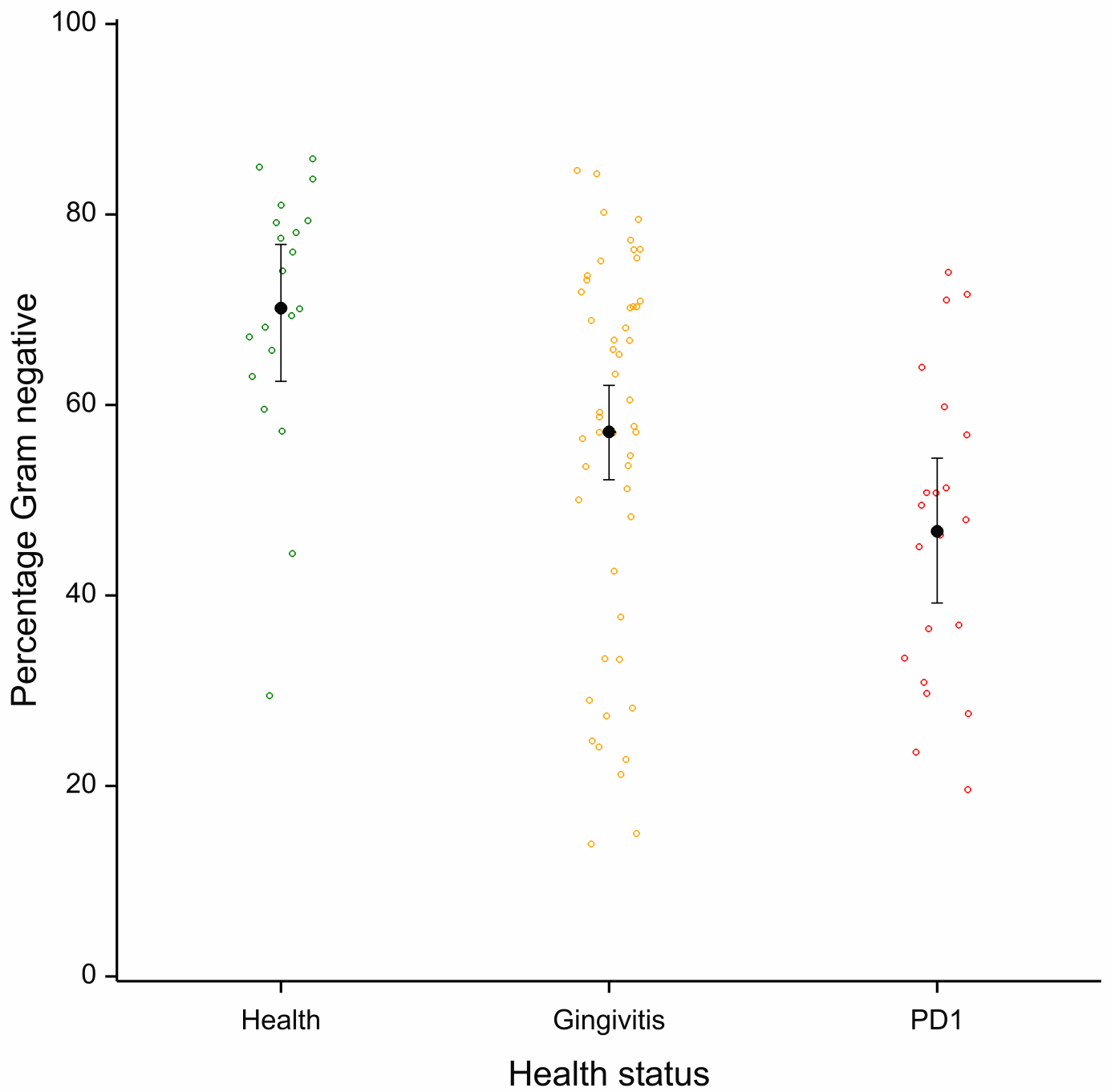

Supplement: S2 Fig — Black bars indicate mean percentage of OTUs that are Gram negative with 95% confidence intervals. (PDF) [file pone.0136986.s002.pdf]

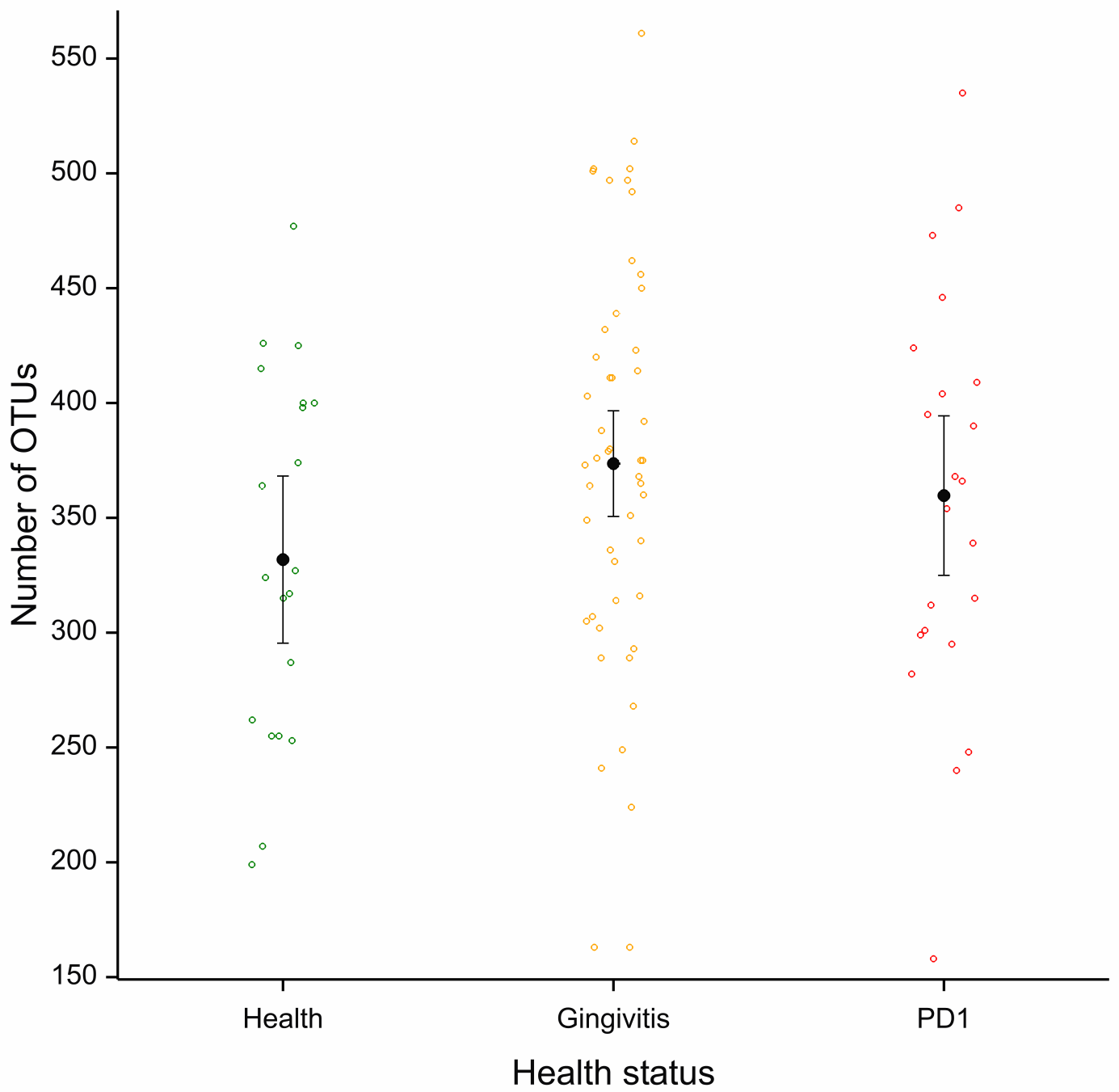

Supplement: S3 Fig — Black bars indicate mean number of OTUs with 95% confidence intervals. (PDF) [file pone.0136986.s003.pdf]

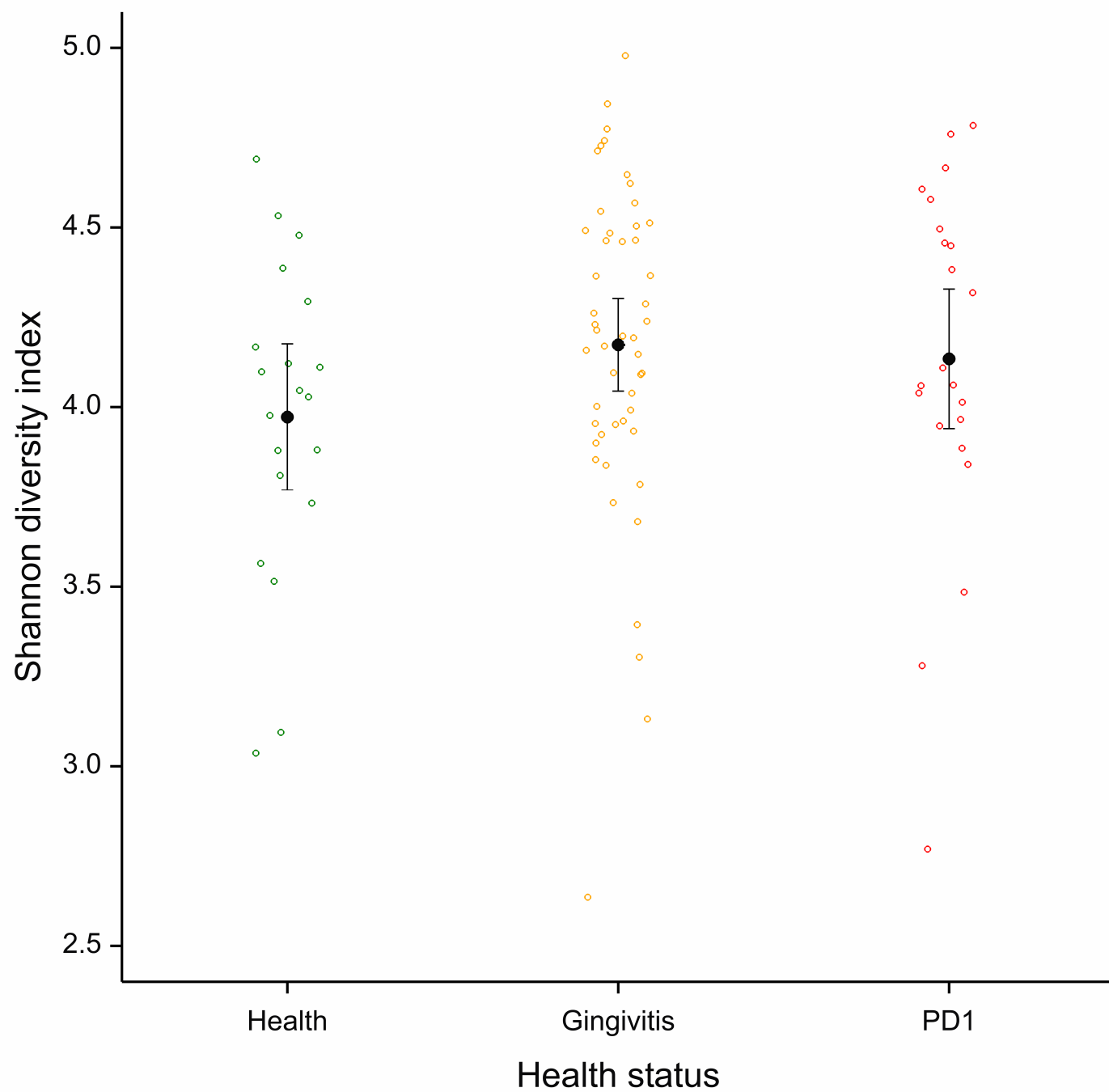

Supplement: S4 Fig — Black bars indicate mean Shannon diversity index for OTUs in that health state with 95% confidence intervals. (PDF) [file pone.0136986.s004.pdf]
